# Supplementary material for: Targeted CRISPR screens reveal genes essential for Cryptosporidium survival in the host intestine
Source: Nat Commun. 2025 Aug 20;16:7749. doi: 10.1038/s41467-025-63012-1 (PMC12368253; doi:10.1038/s41467-025-63012-1)
Supplement: Supplementary file 1 — Supplementary Information [file 41467_2025_63012_MOESM1_ESM.pdf]

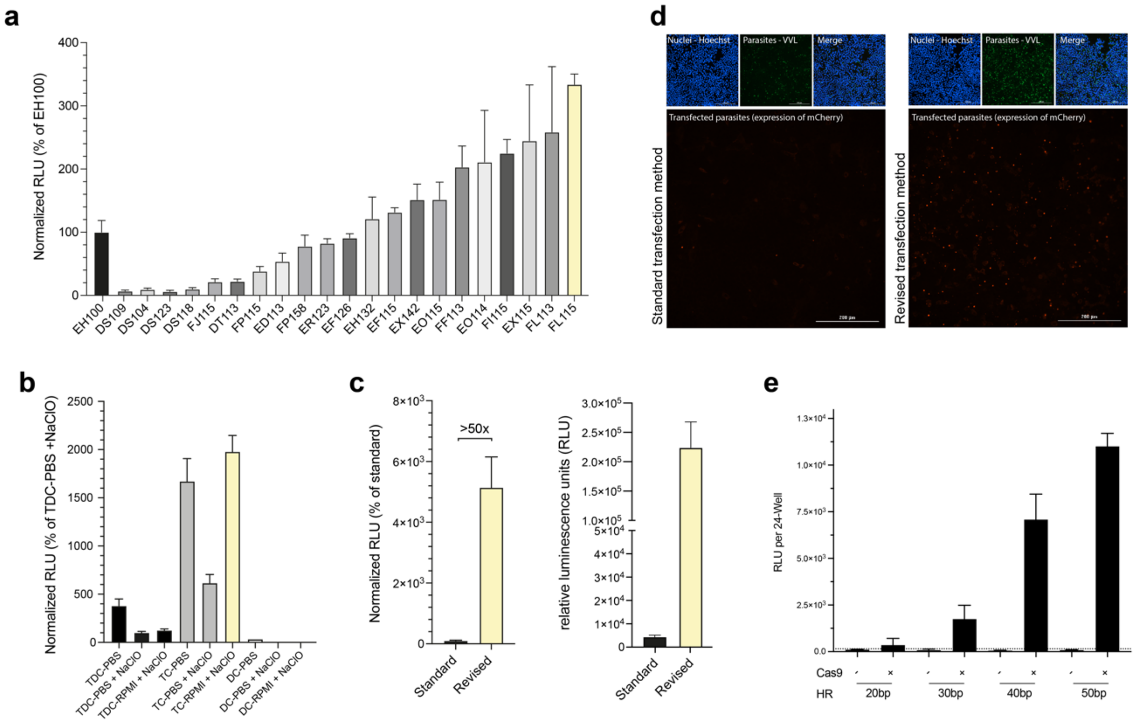

Supplementary Figure 1. Advancement to CRISPR Screening. a-b. Relative luminescence units (RLU) from a HCT8 cell monolayer infected for 24 hours with *C. parvum* parasites that were transiently transfected with a nanoluciferase-mCherry expression vector, trialling multiple electroporation programs (**a**) and excystation reagents used prior to transfection (**b**). TDC (taurodeoxycholate), NaClO (sodium hypochlorite), TC (taurocholate), RMPI (media), DC (deoxycholate) PBS (phosphate-buffered saline). **c**. Quantification of the improved transfection efficiency measured by RLU. In black, the standard method, in yellow, the revised method. Data shows the mean ± standard deviation from 2 biological replicates, graph on left is has been normalised to control and graph on right is the raw relative luminescence readings.**d.** Visualisation of the increased transfection efficiency of the revised method (right panel) compared to the standard method (left panel) using the mCherry-expression vector. Blue (Hoechst), nuclei; green (Vicia villosa lectin (VVL)), parasites; red (mCherry Ab), mCherry/transfected parasites. **e.** RLU from a HCT8 cell monolayer infected for 24 hours with *C. parvum* parasites transfected with a repair cassette designed to replace the thymidine kinase gene, using different lengths of homology repair. Data shows the mean ± sd from three technical replicates.

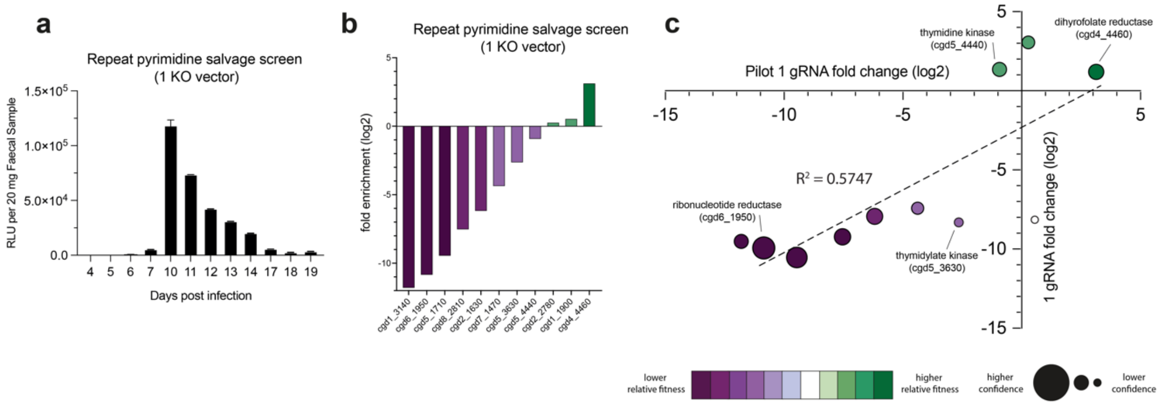

Supplementary Figure 2. Repeat of pyrimidine salvage screen. a. Mouse faecal material was collected and luminescence was monitored during infection (1 KO vector per gene). Data shows the mean faecal luminescence from a pooled cage sample (± SEM of 2 technical replicates), n=4 Ifnγ-/- mice. **b.** Rank ordered fold enrichment scores from the vaccine candidate CRISPR screens. The colour indicates the relative fitness contribution of a gene, with dark purple being high fitness conferring and dark green being low fitness conferring. **c.** Comparison of the fold enrichment scores between the replicate CRISPR screens for each gene. Confidence refers to the inverse of the 95% confidence interval when comparing the log2 fold change scores from each screen (see methods).

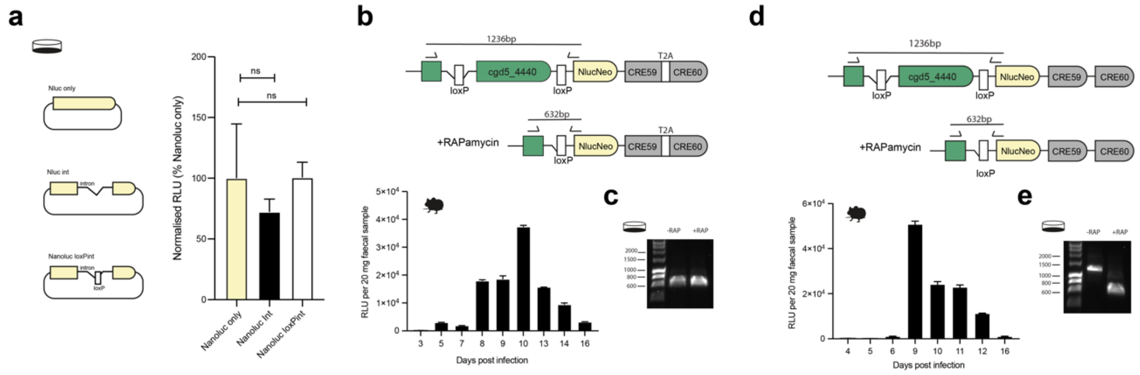

Supplementary Figure 3. Refining diCRE Mediated Excision. a. Schematics of disruption of a nanoluciferase (Nluc) gene mid-codon with a HAP2 (cgd8_2220) intron (Nluc int) or with a HAP2 intron containing a loxP sequence (Nluc loxPint). Vectors were transiently transfected into *C. parvum* sporozoites that were allowed to infect an HCT8 monolayer for 24 hours. Data shown is the mean of 2 biological replicates ± sd of 4 technical replicates. Significance was determined using a one-way ANOVA, ns = not significant **b.** Schematic of the TK-T2A-diCRE parasite line and the corresponding luminescence from mouse faecal material when generating these parasites. Data shown is a biological replicate ± SEM, n=4 Ifn$\gamma^{-/-}$ mice. **c.** Diagnostic PCR to determine the level of excision occurring in the TK-T2A-diCRE parasite line after 24 hours infection of a HCT8 cell monolayer during treatment with rapamycin. **d.** Schematic of the TK-diCRE parasite line and the corresponding luminescence from mouse faecal material when generating the parasites. Data shown is a biological replicate ± SEM, n=4 Ifn$\gamma^{-/-}$ mice. **e.** Diagnostic PCR to determine the level of excision occurring in the TK-diCRE parasite line after 24 hours infection of a HCT8 cell monolayer during treatment with rapamycin. Note that a cell culture dish in the figure indicates an in vitro experiment, while a mouse silhouette indicates an in vivo experiment.

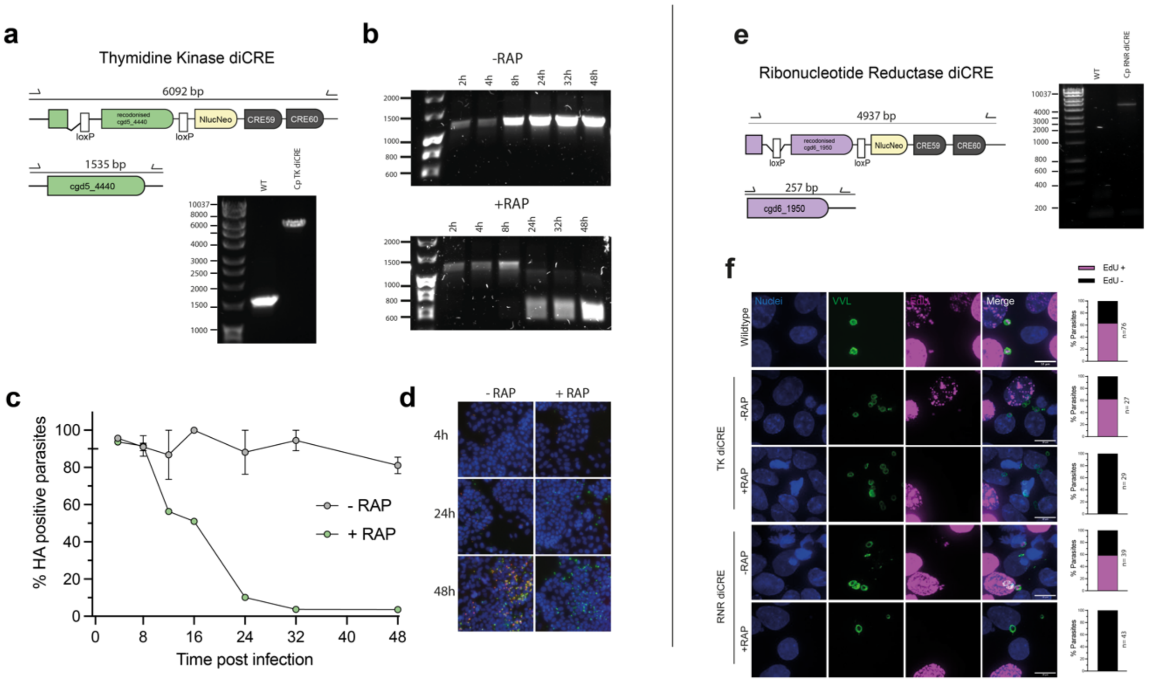

Supplementary Figure 4. diCRE Mediated Excision of TK and RNR. a. Schematic and genotyping PCR of TK-diCRE parasites. **b.** TK-diCRE parasites were allowed to infect a HCT8 monolayer and genomic DNA was extracted at 2, 4, 8, 24, 32 and 48 hours post infection in the presence and absence of rapamycin. Diagnostic PCRs confirmed the level of excision that had occurred at the respective timepoint. **c-d.** TK-diCRE parasites were allowed to infect a HCT8 monolayer in the presence and absence of rapamycin, and the monolayer was fixed and stained at 4, 8, 12, 16, 24, 32 and 48 hours post infection. The HA tag per parasite was quantified by automated imaging, representation images are shown in **d**; blue (Hoechst); green (Vicia villosa lectin (VVL)); red (αHA). Data shown is representative of 2 biological replicates, each with 2 technical replicates ± sd. **e.** Schematic and genotyping PCR of RNR-diCRE parasites. **f.** EdU assay with quantifications showing newly synthesised DNA between 28 and 32 hours in wildtype, TK- and RNR-diCRE parasites in the presence and absence of rapamycin. Blue (Hoechst); green, (Vicia villosa lectin (VVL)); magenta, (EdU). Data shown is two biological replicates with the number of parasites quantified indicated. Scale bar = 10μm.

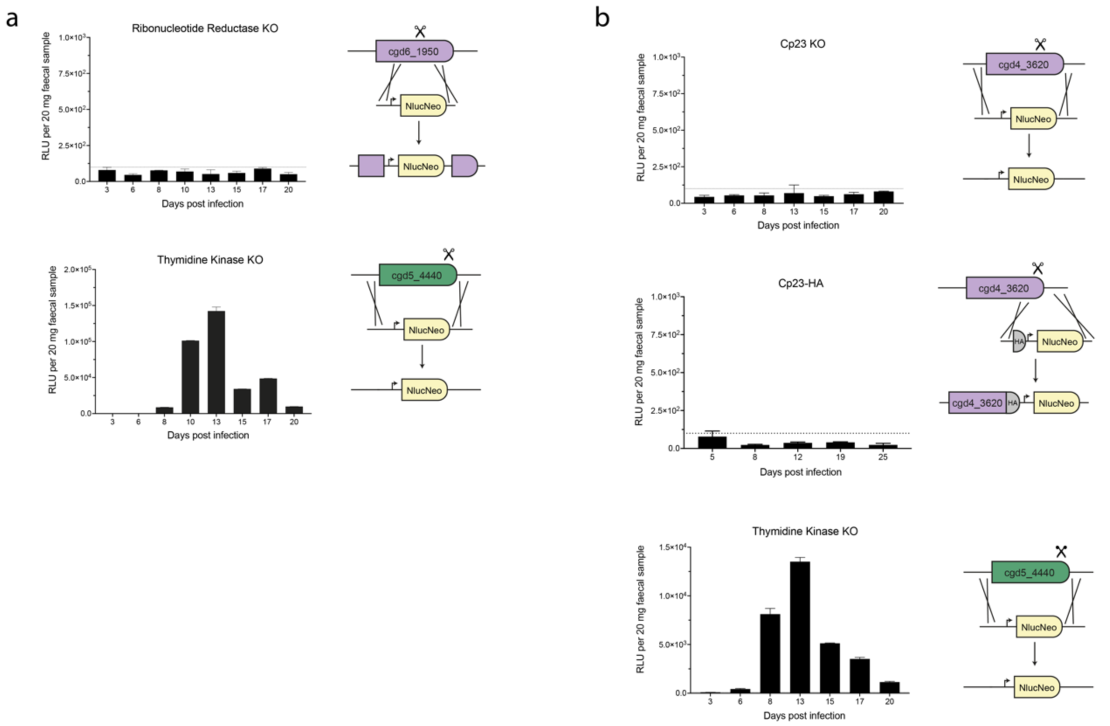

Supplementary Figure 5. Attempts to derive ribonucleotide reductase and Cp23 deficient parasite lines a. Schematic of the attempted ablation of the ribonucleotide reductase (cgd6_1950) endogenous locus by Cas9-driven homologous recombination and the resulting time course of faecal luminescence from a pooled cage sample. As a positive control, the thymidine kinase was targeted for knockout and transfected at the same time as the failed attempt. b. Schematic of the attempted ablation and epitope tagging of the Cp23 (cgd4_3620) endogenous locus by Cas9-driven homologous recombination and the resulting time course of faecal luminescence from a pooled cage sample. As a positive control, the thymidine kinase was targeted for knockout and transfected at the same time as the failed attempts.

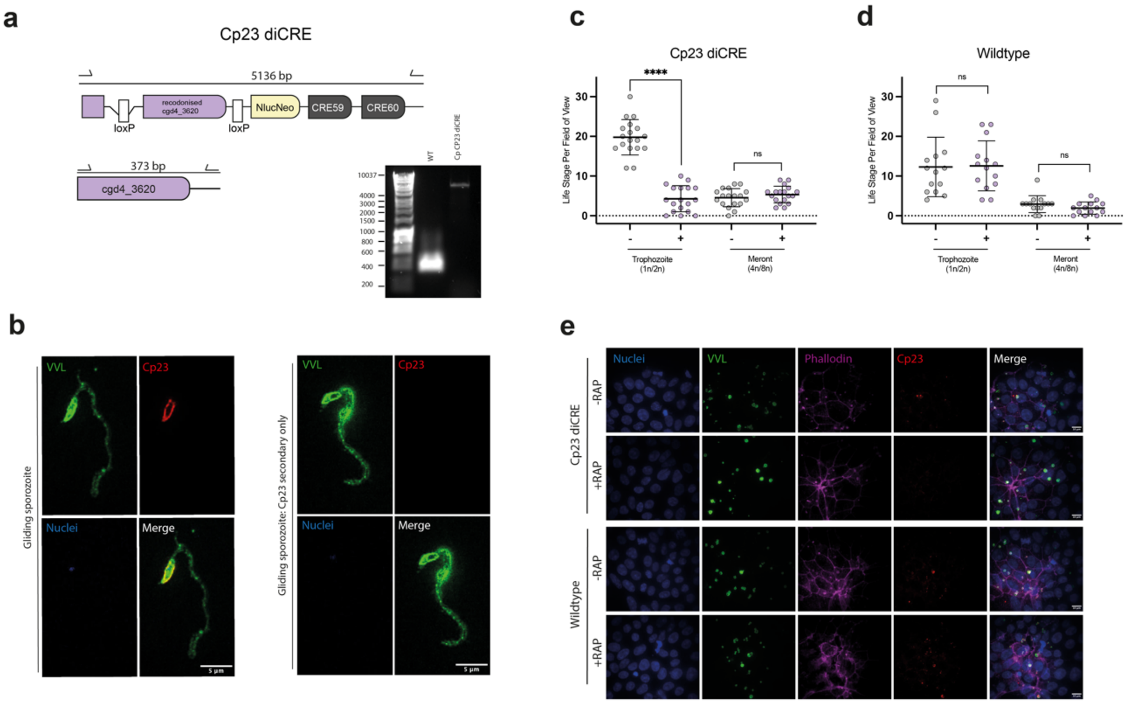

Supplementary Figure 6. Immunodominant Antigen 23 is Essential and Required for Reinvasion of Host Cells. a. Schematic and genotyping PCR of Cp23-diCRE parasites. **b.** Visualising gliding *C. parvum* sporozoites using super-resolution microscopy. Green (Vicia villosa lectin (VVL) which marks both parasite and trail); red (αCp23); blue (Hoechst). Scale bar = 5 μm. **c-e.** Cp23-diCRE and wildtype parasites were allowed to infect an HCT8 monolayer in the presence or absence of rapamycin. At 22 hours post infection the life cycle stage was quantified. Data shows 2 biological replicates. Significance was determined using a two-tailed unpaired t-test, ns = not significant, **** = $p \leq 0.0001$. Representative images are shown in **e**. Blue (Hoechst); green (Vicia villosa lectin (VVL)); magenta (phallodin - actin); red (αCp23). Scale bar = 10μm.

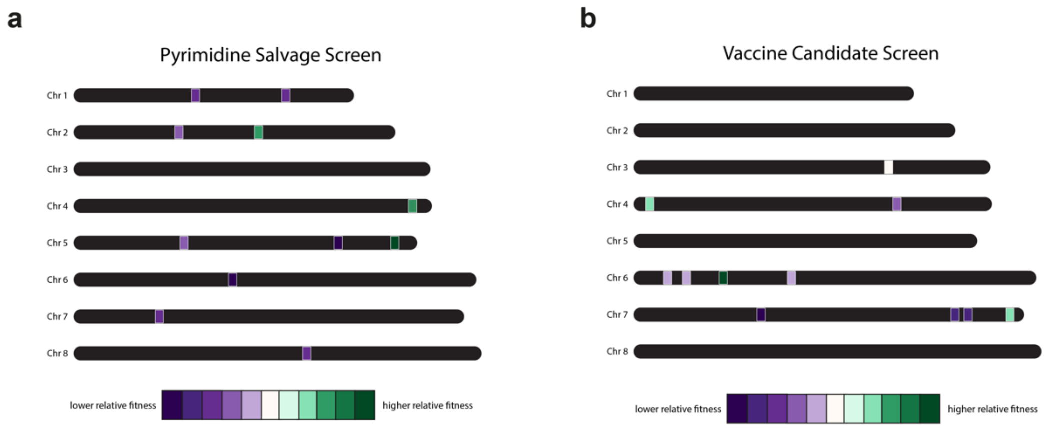

Supplementary Figure 7. Chromosome Locations of the Genes Knocked Out During CRISPR Screens. a-b. Locations of the genes from the pyrimidine salvage (**a**) and vaccine candidate (**b**) CRISPR screens. The colour indicates the relative fitness of the gene knocked out with dark purple being a highly fitness conferring gene and dark green being a low fitness conferring gene.

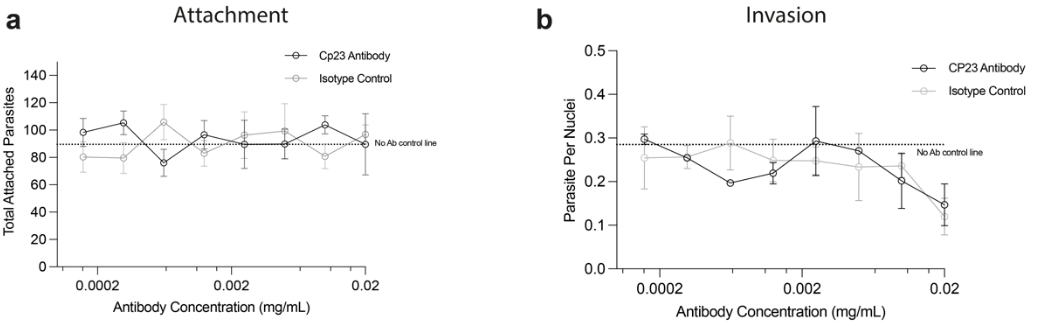

Supplementary Figure 8. Immunodominant Antigen 23 Antibody Does Not Neutralise Infection *In Vitro*. a. Quantifying the number of attached sporozoites on a poly-D treated surface when incubated with the Cp23 antibody or isotype control antibody dilution series. Data shows the mean ± sd of 4 technical replicates. The black line shows Cp23 antibody incubations, the grey line shows isotype control antibody incubations, and the dotted line shows no antibody control incubations. **b.** Quantification of invasion (parasite per host nuclei) when cell monolayers are infected in the presence of the Cp23 antibody or isotype control. The data shows the mean and ± sd of the 4 technical replicates. The back line shows Cp23 antibody incubations, the grey line shows isotype control antibody incubations, and the dotted line shows no antibody control incubations.

Sup Table 1. Genes in the Pyrimidine Salvage CRISPR Screen

Gene ID	Name	Gene Fitness Contribution
cgd1_1900	Fur1p like uracil phosphoribosyltransferase (UPRT)	*Dispensable but fitness conferring* (Kimball et al., 2024)
cgd1_3140	Adenylate kinase/UMP-CMP kinase	
cgd2_1630	Cytidine and deoxycytidylate deaminase family	
cgd2_2780	dCMP deaminase	
cgd4_4460	Bifunctional dihydrofolate reductase/thymidylate synthase (DHFR)	*Dispensable* (Pawlowic et al., 2019)
cgd5_1710	CTP synthase	
cgd5_3630	Thymidylate kinase	
cgd5_4440	Thymidine kinase (TK)	*Dispensable* (Vinayak et al., 2015)
cgd6_1950	Ribonucleotide reductase (RNR)	
cgd7_1470	Cytidine and deoxycytidylate deaminase zinc-binding domain containing protein	
cgd8_2810	Phosphoribulokinase/uridine kinase/Uracil phosphoribosyltransferase	

Sup Table 2. Genes in the Vaccine Candidate CRISPR Screen

Gene ID	Name	Gene Fitness Contributions
cgd3_3370	Uncharacterized protein	
cgd4_32	Apical glycoprotein 1 (AGP1)	*Dispensable* (Akey et al., 2023)
cgd4_3620	Immunodominant antigen 23393226 (Cp23)	
cgd6_1080	Glycoprotein GP40 (GP60)	*Dispensable, but fitness conferring* (Li et al., 2024)
cgd6_1660	Uncharacterized protein with Thrombospondin type-1 (TSP1) repeat (TSP11)	
cgd6_2330	Uncharacterized protein	
cgd6_780	Thrombospondin type-1 (TSP1) repeat/EGF-like domain containing protein (TSP8/MIC1)	
cgd7_1960	WD40/YVTN repeat-like+signal peptide-containing protein	
cgd7_4020	Cryptopsporidial mucin (GP900)	
cgd7_4330	Apical glycoprotein 2 (AGP2)	*Likely Essential* (Akey et al., 2023)
cgd7_5520	Hemogen	

Sup Table 3. Homology Arms/gRNAs used in failed attempts to KO/epitope tag ribonucleotide reductase and Cp23

Gene	guideRNA	Forward Primer	Reverse Primer
cgd6_1950	GATCTACTGTAAA	TGCTAACTTTGCAAAGTTATCATCTATGCATTTTTATGGTTGGAAAA	GTCGTTTGTTAGTCTCCAGTGAATCCTTTTCTTTAGTTTCCGATTTC
(CpRNR) KO	TTGTATG	AAGTGGGGAAACTAAATATACTGAAATTCGGTAGA	AGGAGCTCATCGCGTTTAAACTGATTGG
cgd5_4440	GAAGAATACAATT	ATGGCAAAATTATACTTTTACTATTCAGCAATGAATGCTGGAAAAT	GACTCCTTTTTAGGCACTTTCAAGAGGCGCCATAGCTGCGCCA
(CpTK) KO	TCTAAGG	CAACTGGGGAAACTAAATATACTGAAATTCGGTAGA	AATTTTGAGCTCATCGCGTTTAAACTGATTGG
cgd4_3620	AAAGAAGGCTCA	CTTCCACTTGAAAGAAAAAGTATTTTTAGTTTTATTATTCAATATTAA	GTTAAGTACTCTCTGAGTGTACAACTGCTAAGGCATTTTTTAACAA
(Cp23) KO	ATTAGCCA	AATGGGGAAACTAAATATACTGAAATTCGGTAGA	TAATAGCTCATCGCGTTTAAACTGATTGG
cgd4_3620	ACTCAGAGAGTA	AAGCTCCAGCTGCTGAACCTGCTGCTCAACAAGACAAGCCAG	AAGTATACAAGGGAACTCCCAGTTTAATTTTTCAATTGTGAAAAGT
(Cp23) HA-tag	CTTAACAT	CTGATGCCCCTAGGTACCCGTACGACGTCC	TCAAGTTTCCCCAGGCGCGC
cgd4_3620	AAAGAAGGCT	ATAAATCTGCAGCAGATGCTAACAAACAAAGAGAATTAGCTGAA	CTGGCTTTTGTTGAGCTTGGTTGCTGATTGGAGCTGGATTCTTTA
(Cp23) diCRE	CAATTAGCCA	AAGAAG	CAGCCCTCATCGCGTTTAAACTGATTGG
cgd6_1950	GATCTACTGTAAA	GTCGTTTGTTAGTCTCCAGTGAATCCTTTTCTTTAGTTTCCGATTTC	TGCTAACTTTGCAAAGTTATCATCTATGCATTTTTATGGTTGGAAAA
(CpRNR) diCRE	TTGTATG	AGGGCTCATCGCGTTTAAACTGATTGG	AAGGTAGGTTTGTATTTAATTCATAACTTCG
cgd5_4440	GAAGAATACAATT	TCACTCCTGATTTAAAACTACTTGATATTATTAATCAAGAAAATAATA	TTTTTAGGCACTTTCAAGAGGCGCCATAGCTGCGCCAAATTTTG
(CpTK) diCRE (no T2A site present)	TCTAAGG	AGGTAGGTTTGTATTTAATTCATAACTTCG	CCCGCCTTAATTTAATTTACACCATGCTGCCCAACTTC
cgd5_4440	GAAGAATACAATT	ATTCACTCCTGATTTAAAACTACTTGATATTATTAATCAAGAAAATAA	TTTTTAGGCACTTTCAAGAGGCGCCATAGCTGCGCCAAATTTTG
(CpTK) diCRE (one T2A site present)	TCTAAGG	TAAGGTAAGTTTAATATATTACAAATGGAAATAACTTCGTATAGC	CCCGCCTTAATTTAATTTACACCATGCTGCCCAACTTC

Sup Table 4. Homology Arms/gRNAs used in Pyrimidine Salvage 1 KO Vector CRISPR Screen

KO Vector Target	Hom1/gRNA	Hom2
cgd1_1900_319	CTAATAGGATCTGGTGAAGCGATGGAGAATGCGCTTAGATTTGTATGCAG	CAAATTCGTTTTCAGAAGAGTTATTTAATAACACCTTCCCTATTCTACAT
cgd1_3140_313	TCAGTATCTGGAATAATCTTATACCAACCATCTAAATTATTTTGGTTACG	AGATGCAAGAGTATGGTTGGAATGATAAATATTTTTTGATTGATGGTTTT
cgd2_1630_243	AACGTAAGCTATAGAAACTATAAATTAAGTAAATAAACATATATTACTCA	TTAACTTAACTATTAATTTTTCTAAAGCAACAATTTCACAGTGTCTAGTT
cgd2_2780_524	CTCTTCAAAATCTTGAACCAATATTGAGGCAAGTTCTTTTAACTTTCCTG	GAATGCAAATTTTGAAGAAGTTGAAAAATCTATTGAGGTATTAAAAAATT
cgd4_4460_156	TAATTGTGACTCGAATAAGAAGAATGCACTAATTATGGGAAGAAAAACAT	GGAAATAACGACAATTTTTCTATTTTTAAGAGGTCTTCTTCCAATTGAAT
cgd5_1710_235	ATAGATCCTTATTTGAATATTGACGCAGGGACTATGTCTCCCTTTGAGCA	TTCCCAAATCCAAATCGACTTCCTCTCCATCATCTAATACAAAAACCTCA
cgd5_3630_140	ATATGAAATTGGGGGTAAGAAGAGGGGGATATGCTGGCAGAAAGAGAGAA	GAATCCCTCTCCTCCATTAGTAATTTTGACTAACTTTAGATAAAGGAATT
cgd5_4440_332	AGAGAATTATGCATTGTTGTTGATAAGCTAAATATTCCAGTACTATGCTA	ATAAGTATTTACTTCCTTCAAATAAATTTCCCTTAAAGTCTGTTCTCAAA
cgd6_1950_240	TGTGTAGATTAGAAGTCGATATCCGAGCAGCGAGTTTAGAGAAATCATTG	ATCTGAATTGGACGAACTTGCTTCGCAAACTTGTGCATATATGGCAGCTA
cgd7_1470_400	GATAAAGGCCAAATTTTTGAAAGTTGGGAAAACTTTTCCCTAGTTAATGG	AAGAAAACCATATTGATAAAATTTGGAATGTAATAGAGGTTCCAAAGAAT
cgd8_2810_291	TTCTGTGGACTTTGAACTTCTATACAATGTTTTACTAAGTTTGAAAAACG	TTCCAATCTCTTATGTTGTTTAAAACAGTAGTTAGGGATGTGAACCCCTT

Sup Table 5. Homology Arms/gRNAs used in Pyrimidine Salvage 2 KO Vector CRISPR Screen

KO Vector Target	Hom1/gRNA	Hom2
cgd1_1900_232	CTTCCTTATGATTATAAGGAAATTAAAACCCCAAATGGAATCGAAGTCAA	CACCAGATCCTATTAGGCTCACGCCGCAGATGGGAGTGTTAAATGCAATT
cgd1_3140_162	AATGTCTAGAAAGGATGAAACCAGCGAGTTAATTGACAGTTATATCAGAG	CATCTTCTTCTTTAACAAACCAACAGTAATCTCAACAGGAACAATTAATC
cgd2_1630_37	TATAACAATGAAGAGTTAGAGATGTTTATGAAAAGAGCGATTGAACTAGT	TCTGACTAAAAATAATAAATTATTACAGCTTTACAAAGATATTAGCTTTA
cgd2_2780_222	ATGTGGCAAAAAAGCAATAAGACAAATTTGCTTTTGCGGAAGCGATTCAG	TATCATGAAAGCTTTCTTTGCTCTGAACCTAATACAATCATCATAAGATT
cgd4_4460_9	TGTGAATTTCAGAACTTTTAAAATGAGTAAAAAGAACGTTTCAATTGTTG	CCAAGGTAATTGTCCGTTAATTCCTATTCCTCTACTCAAAACAGAAGCTG
cgd5_1710_160	CTAGGTAAAGGAATAGCTATAAGCTCGCTTGGCTTATGCCTTAAAAGCAG	CTGCGTCAATATTCAAATAAGGATCTATTTTTATCGCTGTTACATTATAT
cgd5_3630_73	GTATTAGAAGGAACAGATAGGTAAGTTTGTTAAAATTTAAATATTAAGAG	CAGCATATCCCCCTCTTCTTACCCCCAATTTCATATTTATTTCTTCTCCA
cgd5_4440_223	ATTTTTTCCTTATTATTTTCTTGATTAATAATATCAAGTAGTTTTAAATC	GCTCAATTTGCTCTAGAATTGGTCTCTCAGAAAAAGCACACACATTCACT
cgd6_1950_124	CTATATAGACCATTTATCACTGCTTGAGTAACCCTCGCTGGGTCAACAAG	CATTTGACCAGATTCTTAGCAGAATCACTAAATTATCATATGGACTTCAT
cgd7_1470_202	AATTCCAAGTCATTCTCTTTAATAACTACTTTACGCATTCTTTTTATGTG	CTAATGAATTTTCAAGATTTGTTAATAATGACCAGTTTACAAAGAGATTC

cgd8_2810_203	TTACGGTAATTGAGACTGATAGTTTTTATAAAACTCCTGTCTTAGAAGAG	TCAAAGTCCACAGAATTAGGATGATCAAAGTTGTAGTCTGCCATAGTTTG

** Table 3 KO used in addition to Table 4 KO vectors*

Sup Table 6. Homology Arms/gRNAs used in Vaccine Candidate 2 KO Vector CRISPR Screen

KO Vector Target	Hom1/gRNA	Hom2
cgd7-4020_67_revco	ACTCGATTTAAATGCAAGTGAAAAAAGTGGGTTCATAATAACAGCCACAA	GGTGAAGTCAAAAATCATGGTGAACATTAAAGTGAGCTCATCGGCAATAG
cgd7-4020_188_revco	TCTTGCCAGTAGAATCAATAAGCAAGAAAGTTGTTGGGTCTAAATCTGAA	ATTGAATCATCTGGTGCAGTTTCAAATGAAAAATTTGTAATCCCATCTCT
cgd4-32_141_revcom	GATTGCCACGTCAAGTTTAAATCTATTGCATTACTTGCCATTAATTCCTC	GTGGCAAACTTATTGACTCAATAAACGAAAATTATGATAGATTTTCATAT
cgd4-32_293	CTGAACTCTTCAACAAGAGAAGAATTAAAAACAAACTGGAGTTACACTAC	TACCTTCTAATGCATTGATTGTGCACGAAACAGAACTTAGATTTTTGACT
cgd4-3620_84_revcom	CTTTGTTTGTTAGCATCTGCTGCAGATTTATTTTCAGCAACTTTAGTTTC	ATTTTTAGTTTTATTATTCAATATTAAAAATGGGTTGTTCATCATCAAAG
cgd4-3620_183_revcom	GACTTCTTTGGTTCTTCTGGCTTTTGTTGAGCTTGGTTGCTGATTGGAGC	AAAGAGAATTAGCTGAAAAGAAGGCTCAATTAGCCAAGGCTGTAAAGAAT
cgd6-1080_106_revcom	AACATCCTTTAAAGTTCCTCTGAGTGGAACGGCTGGGGCTGAGAATACAG	AAGGAAGATGAGATTGTCGCTCATTATCGTATTACTCTCCGTTATAGTCT
cgd6-1080_632	CAGGATTTCAGCACTCTCTCTGCTAATTCAAGTAGTCCAACTGAAAATGG	TTTCCTCTGAGAGTGATCTTCTTGATCTTGATGAAGCCTGACCCGCAGAT
cgd6-780_211_revcom	AGAATACCAAGGTAGCTTATTTGAACTAAATGTATATACTAACCCTTCAC	AAATTCTTTACCACCAAGTTTCAGTTGGACAAAAGCATGGAAAGATATTA
cgd6-780_347_revcom	TAGATTCCACATCTACTGTTTGTGCTGCAAAATTTATCATCAACTTACAG	GCAGAAAATGATAAAGAAACTTTGGTAACAATTCAGAATGGTGATTTATA
cgd7-4330_329	ACTCCATCAATATCAGGATATAATCGATGCGCTGTAATTATTGGTCTGAG	TTCCTAAAGAGATGTTTTTGGAGCCAAAAGGACTAACATGAATTCTATCT
cgd7-4330_517_revcom	GTTATCAACGTTAGTAATTCCCTGTGATATTGGCGTAGAAACAACTTGAG	CGTACCAGAATATGCCAGATACGGGGCTATTCATTCTGGGTACAGTCCTT
cgd3-3370_100	ATTCCTAGCAGGGTTGTTTCTCAACACCCTTCTGGGATGCGCTTCTGTTT	ACTTTCTCTTGGAACAGATGCAGCTGGAGGAATTTCGGTTTCTGATGATG
cgd3-3370_1241	GGTTCTGCAGTTGGTAGATACAAATGTAGAGTTGGAGAAACGTCTTGTTA	TTTCTACTTTTTGACCGGGAGATAATGGGTTTTGTAGTCTAAATTCAGAA
cgd6-2330_164	GCTAGTTGTTTGAATGCTTCGTACTCATTCATTGAAGGTGGCATGACACC	ATTCTAACGATGCAGATGGTAAAAGAACGACAGAAACACGGATAGCATCG
cgd6-2330_316_revcom	ACCATTCTTGTAAAGTGAATATGCTCTTAATGAAGCTATGCCACTTTCAA	TTCTTCATTCCAGAATATTGCAACATTAAGATTCGGTGTGGACTCATGTT
cgd6-1660_124	ATGCTTGGGGCCAGAGCATGTAGAATTTCATTTGGTCAACGGAAATAAAG	TTTTCTATTTTCAACTTCGAACCTGCCATTTCCTTCTATTTGATTTAAAT
cgd6-1660_998	GTAATAATTTTGAGCGAATCCCGAAATGAAATAAGGCCATACGGCTATAG	CGTTGTTTAAGTCAATATTTTCGATAAATTGAGTCAGTATCATAGGGCTT
cgd7-1960_160	AGCTTCAGAAGCAAAGGTAGAAACAGAAAACCCATTGCTATCAAGTACAG	CATGGAAAGCAACTTATCAGAACAACAGGCACATGGATTGTTAGAACAAT
cgd7-1960_317	TTAAAGAATCTATTTTGCACTTTAGCATGTAACGAGGAGTCATCAGACAT	AACATCCTGACTCGCATACAGACAAGACCATAATTTCATTTGACAATGTT
cgd7-5520_1216_revco	CGCTAAGTCTTTCTCAGAACCAAAAGTCATTTTATTCTCAGCGCGATTAA	TTTCTATACAAAAACATGTTTTGAAAAGAATGAAGCACATTGTCTTAAAC
cgd7-5520_1115_revco	AAACACAAGCGATTGAATAACTTACAGCTTCAAGAGCGTGTTTCCTATCA	TTTGAGCTTGGCTTGATAAATGGTAGTTGGCTCGGAGGTGATATTTTTAT

Sup Table 7. List of Antibodies Used in this Work

Antibody	Dilution factor (IFA or Expansion*)	Source	Catalogue
mCherry Monoclonal Antibody (16D7)	1:1000	Invitrogen	M11217
Anti-*Cryptosporidium* Immunodominant Antigen Cp23	1:1000 or 1:250*	Stratech	LS-C137378 Lot #234986
Vicia villosa lectin (VVL)	1:4000	2B Scientific	FL-1231-2
Lectin HPA AF488	1:5000 or 1:1000*	Invitrogen	L11271
HPA-Alexa 647	1:5000	Thermo Fischer	L32454
Hoechst 33342	1:10,000	Invitrogen	H3570
Goat anti-Rabbit AF 647	1:1000	Invitrogen	A-21245
Goat anti-Mouse AF546	1:1000 or 1:250*	Invitrogen	A-11030
Goat anti-Rat AF647	1:1000	Invitrogen	A-21247

Alexa Fluor™ 405 NHS Ester	1:250*	Thermo Scientific	A30000
SYTOX Deep Red Nuclei Acid Stain	1:1000*	Invitrogen	S11381
Anti-TrpB	1:1000	Kind gift from lab of Boris Striepen	Generated towards TrpB protein
Click-IT Plus EdU Cell Proliferation Kit, Alexa Fluor 647	10mM	Thermo Fischer	C10340
