# Supplementary material for: Targeted CRISPR screens reveal genes essential for Cryptosporidium survival in the host intestine
Source: Nat Commun. 2025 Aug 20;16:7749. doi: 10.1038/s41467-025-63012-1 (PMC12368253; doi:10.1038/s41467-025-63012-1)
Supplement: Supplementary file 2 — Reporting summary [file 41467_2025_63012_MOESM2_ESM.pdf]

nature portfolio

Corresponding author(s): Adam Sateriale

Last updated by author(s): Jul 30, 2025

Reporting Summary

Nature Portfolio wishes to improve the reproducibility of the work that we publish. This form provides structure for consistency and transparency in reporting. For further information on Nature Portfolio policies, see our Editorial Policies and the Editorial Policy Checklist.

Statistics

For all statistical analyses, confirm that the following items are present in the figure legend, table legend, main text, or Methods section.

n/a	Confirmed	
☐	☒	The exact sample size (*n*) for each experimental group/condition, given as a discrete number and unit of measurement
☐	☒	A statement on whether measurements were taken from distinct samples or whether the same sample was measured repeatedly
☐	☒	The statistical test(s) used AND whether they are one- or two-sided<br>*Only common tests should be described solely by name; describe more complex techniques in the Methods section.*
☐	☒	A description of all covariates tested
☒	☐	A description of any assumptions or corrections, such as tests of normality and adjustment for multiple comparisons
☐	☒	A full description of the statistical parameters including central tendency (e.g. means) or other basic estimates (e.g. regression coefficient) AND variation (e.g. standard deviation) or associated estimates of uncertainty (e.g. confidence intervals)
☐	☒	For null hypothesis testing, the test statistic (e.g. *F*, *t*, *r*) with confidence intervals, effect sizes, degrees of freedom and *P* value noted<br>*Give P values as exact values whenever suitable.*
☒	☐	For Bayesian analysis, information on the choice of priors and Markov chain Monte Carlo settings
☒	☐	For hierarchical and complex designs, identification of the appropriate level for tests and full reporting of outcomes
☒	☐	Estimates of effect sizes (e.g. Cohen's *d*, Pearson's *r*), indicating how they were calculated

Our web collection on statistics for biologists contains articles on many of the points above.

Software and code

Policy information about availability of computer code

Data collection	N/A
Data analysis	Microsoft Excel for Mac. Version 16.95.4 (25040241).<br>GraphPad Prism 10 for macOS. Version 10.3.0 (461).

For manuscripts utilizing custom algorithms or software that are central to the research but not yet described in published literature, software must be made available to editors and reviewers. We strongly encourage code deposition in a community repository (e.g. GitHub). See the Nature Portfolio guidelines for submitting code & software for further information.

Data

Policy information about availability of data

All manuscripts must include a data availability statement. This statement should provide the following information, where applicable:

- Accession codes, unique identifiers, or web links for publicly available datasets
- A description of any restrictions on data availability
- For clinical datasets or third party data, please ensure that the statement adheres to our policy

All raw data to create used to create graphs have been made available as a Source Data file.

Research involving human participants, their data, or biological material

Policy information about studies with human participants or human data. See also policy information about sex, gender (identity/presentation), and sexual orientation and race, ethnicity and racism.

Reporting on sex and gender	N/A
Reporting on race, ethnicity, or other socially relevant groupings	N/A
Population characteristics	N/A
Recruitment	N/A
Ethics oversight	N/A

Note that full information on the approval of the study protocol must also be provided in the manuscript.

Field-specific reporting

Please select the one below that is the best fit for your research. If you are not sure, read the appropriate sections before making your selection.

☒ Life sciences ☐ Behavioural & social sciences ☐ Ecological, evolutionary & environmental sciences

For a reference copy of the document with all sections, see nature.com/documents/nr-reporting-summary-flat.pdf

Life sciences study design

All studies must disclose on these points even when the disclosure is negative.

Sample size	*Describe how sample size was determined, detailing any statistical methods used to predetermine sample size OR if no sample-size calculation was performed, describe how sample sizes were chosen and provide a rationale for why these sample sizes are sufficient.*
Data exclusions	No data has been excluded from the analysis.
Replication	Two or three biological replicates, each with at least two technical replicates, were carried out.
Randomization	Litter mates of either sex were randomly used for experimental groups.
Blinding	Microscopy and luminescence based in vitro work was blinded. Animal work was not blinded.

Reporting for specific materials, systems and methods

We require information from authors about some types of materials, experimental systems and methods used in many studies. Here, indicate whether each material, system or method listed is relevant to your study. If you are not sure if a list item applies to your research, read the appropriate section before selecting a response.

Materials & experimental systems

n/a	Involved in the study	
☐	☒	Antibodies
☐	☒	Eukaryotic cell lines
☒	☐	Palaeontology and archaeology
☐	☒	Animals and other organisms
☒	☐	Clinical data
☒	☐	Dual use research of concern
☒	☐	Plants

Methods

n/a	Involved in the study	
☒	☐	ChIP-seq
☒	☐	Flow cytometry
☒	☐	MRI-based neuroimaging

Antibodies

Antibodies used	(1) mCherry Monoclonal Antibody (16D7) (Invitrogen, M11217), (2) Anti-Cryptosporidium Immunodominant Antigen Cp23 (Stratech, LS-C137378, Lot #234986), (3) Vicia villosa lectin (VVL) (2B Scientific, FL-1231-2), (4) Lectin HPA AF488 (Invitrogen, L11271), (5) HPA-Alexa 647 (Thermo Fischer, L32454), (6) Hoechst 33342 (Invitrogen, H3570), (7) Goat anti-Rabbit AF

	647 (Invitrogen, A-21245), (8) Goat anti-Mouse AF546 (Invitrogen, A-11030), (9) Goat anti-Rat AF647 (Invitrogen, A-21247), (10) Alexa Fluor™ 405 NHS Ester (Thermo Scientific, A30000), (11) SYTOX Deep Red Nuclei Acid Stain (Invitrogen, S11381), (12) Anti-TrpB (generated towards TrpB protein, kind gift from Boris Striepen), (13) Click-IT Plus EdU Cell Proliferation Kit, Alexa Fluor 647 (Thermo Fischer, C10340).
Validation	The Anti-Cryptosporidium Immunodominant Antigen Cp23 primary antibody was validated by inducible knockout.

Eukaryotic cell lines

Policy information about cell lines and Sex and Gender in Research

Cell line source(s)	HCT8 cells were obtained from the ATCC and maintained by Cell Services at The Francis Crick Institute.
Authentication	*Describe the authentication procedures for each cell line used OR declare that none of the cell lines used were authenticated.*
Mycoplasma contamination	HCT8 cells were routinely tested for mycoplasma contamination. All test results were negative.
Commonly misidentified lines (See ICLAC register)	*Name any commonly misidentified cell lines used in the study and provide a rationale for their use.*

Animals and other research organisms

Policy information about studies involving animals; ARRIVE guidelines recommended for reporting animal research, and Sex and Gender in Research

Laboratory animals	Interferon gamma deficient (Ifng-/-) mice between 3-8 weeks of age were used.
Wild animals	The study did not involve wild animals.
Reporting on sex	Litter mates of either sex were randomly used for experimental groups.
Field-collected samples	The study did not involve collecting samples from the field.
Ethics oversight	All experiments involving mice were done under the care and supervision of the Francis Crick Institute veterinary and Biological Research Facility under protocols approved under project license PP8575470.

Note that full information on the approval of the study protocol must also be provided in the manuscript.

Plants

Seed stocks	N/A
Novel plant genotypes	N/A
Authentication	N/A
