# Supplementary material for: Targeted CRISPR screens reveal genes essential for Cryptosporidium survival in the host intestine
Source: Nat Commun. 2025 Aug 20;16:7749. doi: 10.1038/s41467-025-63012-1 (PMC12368253; doi:10.1038/s41467-025-63012-1)
Supplement: Supplementary file 7 — Description Of Additional Supplementary File [file 41467_2025_63012_MOESM7_ESM.pdf]

Description of Additional supplementary files

Supplementary Movie 1:

Live imaging of Cryptosporidium parasite egress was carried out at 18 hours post infection of an intestinal epithelial (HCT8) monolayer infected with Cp23-diCRE parasites. Here, parasites are treated with DMSO vehicle control.

Supplementary Movie 2:

Live imaging of Cryptosporidium parasite egress was carried out at 18 hours post infection of an intestinal epithelial (HCT8) monolayer infected with Cp23-diCRE parasites. Here, parasites are treated with DMSO vehicle control.

Supplementary Movie 3:

Live imaging of Cryptosporidium parasite egress was carried out at 18 hours post infection of an intestinal epithelial (HCT8) monolayer infected with Cp23-diCRE parasites. Here, parasites are treated with rapamycin, causing excision of the Cp23 gene.

Supplementary Movie 4:

Live imaging of Cryptosporidium parasite egress was carried out at 18 hours post infection of an intestinal epithelial (HCT8) monolayer infected with Cp23-diCRE parasites. Here, parasites are treated with rapamycin, causing excision of the Cp23 gene.
